# Supplementary material for: Inequalities in COVID-19 severe morbidity and mortality by country of birth in Sweden
Source: Nat Commun. 2023 Aug 15;14:4919. doi: 10.1038/s41467-023-40568-4 (PMC10427621; doi:10.1038/s41467-023-40568-4)
Supplement: Supplementary file 1 — Supplementary Information [file 41467_2023_40568_MOESM1_ESM.pdf]

Supplementary Information

Supplementary Figure 1: Seven day rolling average of COVID-19 related mortality. The dotted lines show the breaks used in splitting the data. The waves are defined by elevated levels of COVID-19 related mortality and the times between the waves are referred to as phases 1,2,3.

Supplementary Figure 2: Seven day rolling average of ICU admission. The dotted lines show the breaks used in splitting the data. The waves are defined by elevated levels of COVID-19 related ICU admissions and the times between the waves are referred to as phases 1,2,3.

Supplementary Table 1: Association between country/region of origin and Covid-19 related ICU admission: RRs and 95% Cis by different model specification.

Supplementary Table 2: Association between country/region of origin and Covid-19 related mortality: RRs and 95% Cis by different model specification.

Supplementary Table 3: Percentage change of excess mortality for the subsequent models.

Supplementary Table 4: Association between country/region of origin and Covid-19 related ICU admission for the seven time periods (the four waves of elevated Covid-19 infections and three phases of low Covid-19 infections): RRs and 95% Cis for M1, M4, and M5 model specifications*.

Supplementary Table 5: Association between country/region of origin and Covid-19 related mortality for the seven time periods (the four waves of elevated Covid-19 infections and three phases of low Covid-19 infections): RRs and 95% Cis for M1, M4, and M5 model specifications*.

Supplementary Figure 1: Seven day rolling average of COVID-19 related mortality. The dotted lines show the breaks used in splitting the data. The waves are defined by elevated levels of COVID-19 related mortality and the times between the waves are referred to as phases 1,2,3.

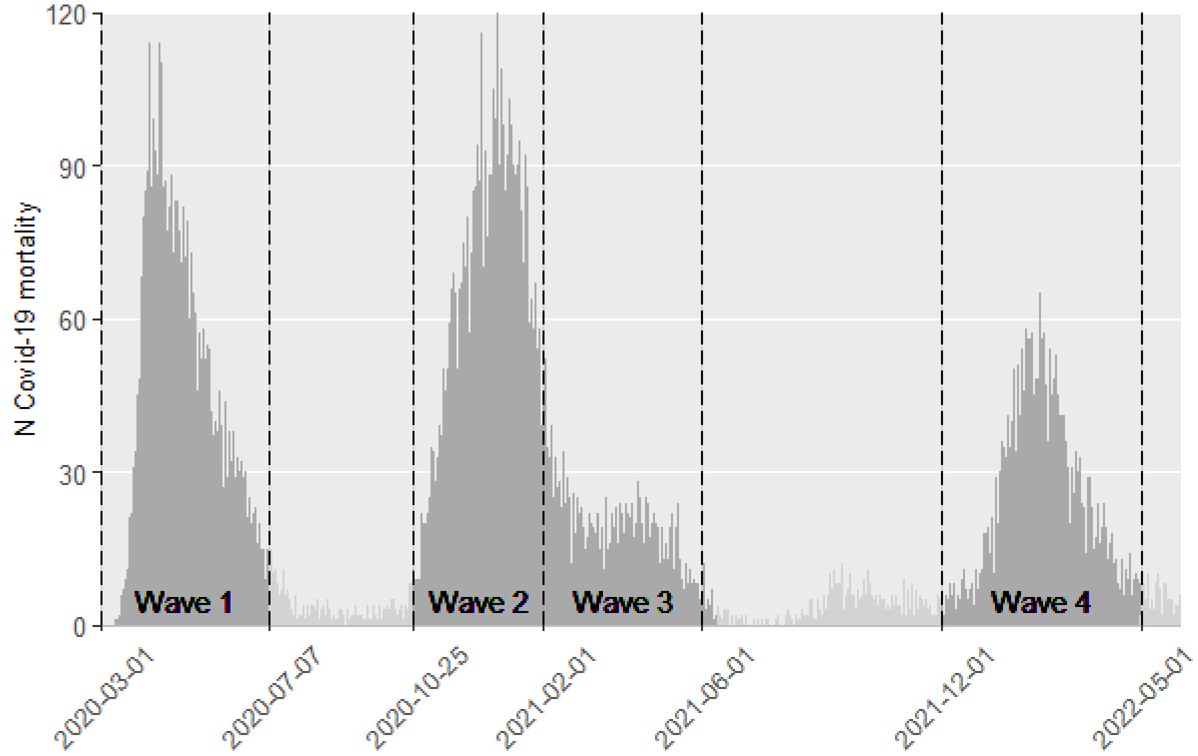

Supplementary Figure 2: Seven day rolling average of ICU admission. The dotted lines show the breaks used in splitting the data. The waves are defined by elevated levels of COVID-19 related ICU admissions and the times between the waves are referred to as phases 1,2,3.

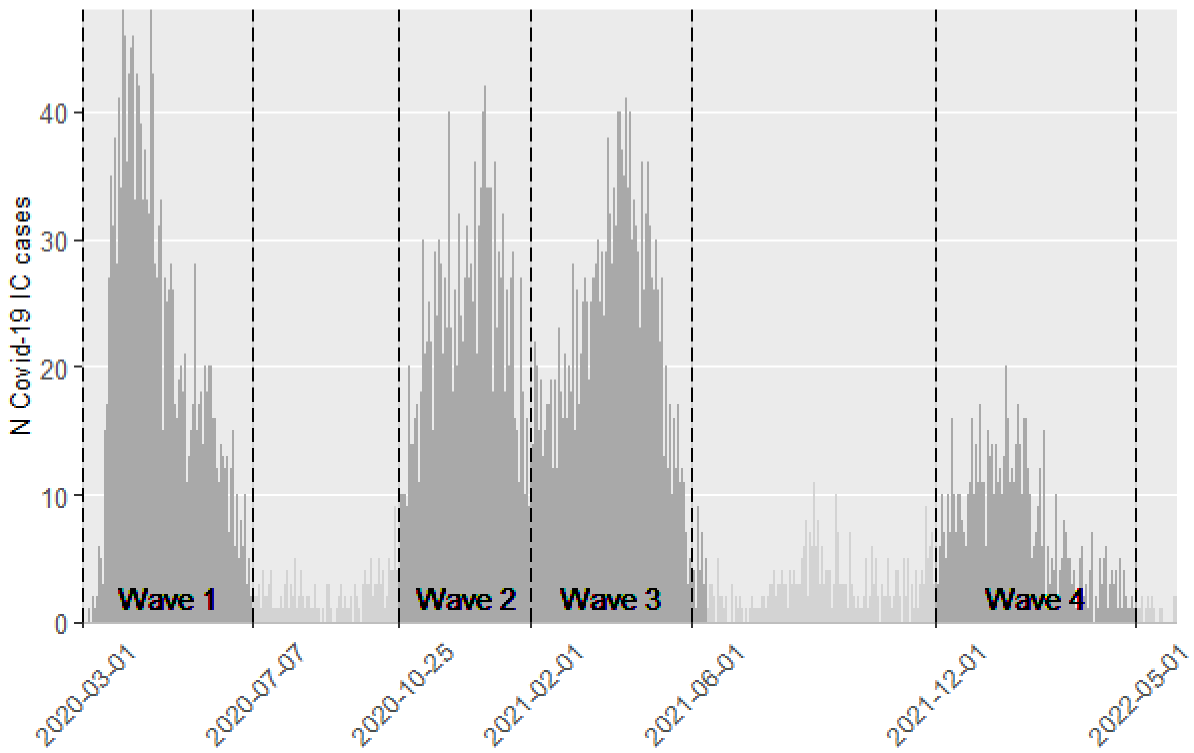

Supplementary Table 1: Association between country/region of origin and Covid-19 related ICU admission: RRs and 95% Cis by different model specification.

	Model 1: Sex, age, and region of origin	Model 2: Model 1 + SES	Model 3: Model 1 + living conditions	Model 4: Model 1 + vaccines	Model 5: Model 1 + SES + living conditions + vaccines
AIC:	149547	148999	148850	148779	147653
Residuals					
Region of Origin	RR (LCL-UCL)	RR (LCL-UCL)	RR (LCL-UCL)	RR (LCL-UCL)	RR (LCL-UCL)
Sweden	1 (ref)	1 (ref)	1 (ref)	1 (ref)	1 (ref)
Nordics wo Sweden	1.58 (1.42-1.75)	1.45 (1.31-1.62)	1.48 (1.33-1.64)	1.52 (1.37-1.69)	1.34 (1.21-1.49)
EU28 wo Nordics	1.57 (1.41-1.74)	1.47 (1.31-1.64)	1.46 (1.32-1.63)	1.38 (1.24-1.53)	1.23 (1.10-1.38)
Europe wo EU28	3.45 (3.17-3.76)	3.01 (2.76-3.28)	3.09 (2.83-3.37)	3.05 (2.81-3.32)	2.47 (2.26-2.70)
Middle East	4.33 (4.04-4.64)	3.67 (3.40-3.95)	3.63 (3.37-3.90)	3.93 (3.67-4.21)	2.97 (2.74-3.21)
Africa	4.12 (3.70-4.60)	3.35 (2.99-3.76)	3.17 (2.83-3.55)	3.68 (3.30-4.10)	2.50 (2.22-2.81)
Asia	3.68 (3.31-4.09)	3.16 (2.83-3.54)	3.17 (2.84-3.53)	3.47 (3.12-3.85)	2.71 (2.42-3.03)
North America	0.73 (0.41-1.28)	0.77 (0.44-1.36)	0.66 (0.37-1.16)	0.68 (0.38-1.19)	0.66 (0.37-1.16)
South America	3.57 (3.09-4.12)	3.31 (2.86-3.83)	2.83 (2.45-3.28)	3.28 (2.84-3.79)	2.50 (2.16-2.90)
Sex	RR (LCL-UCL)	RR (LCL-UCL)	RR (LCL-UCL)	RR (LCL-UCL)	RR (LCL-UCL)
Female	1 (ref)	1 (ref)	1 (ref)	1 (ref)	1 (ref)
Male	2.34 (2.23-2.45)	2.33 (2.22-2.44)	2.39 (2.29-2.51)	2.30 (2.20-2.41)	2.32 (2.22-2.43)
Age	RR (LCL-UCL)	RR (LCL-UCL)	RR (LCL-UCL)	RR (LCL-UCL)	RR (LCL-UCL)
[20,30)	1 (ref)	1 (ref)	1 (ref)	1 (ref)	1 (ref)
[30,35)	1.28 (1.06-1.55)	1.41 (1.17-1.70)	1.28 (1.06-1.54)	1.31 (1.09-1.58)	1.40 (1.16-1.69)
[35,40)	1.59 (1.33-1.90)	1.79 (1.49-2.14)	1.63 (1.36-1.95)	1.66 (1.39-1.98)	1.85 (1.54-2.21)
[40,45)	2.48 (2.11-2.93)	2.79 (2.36-3.30)	2.60 (2.21-3.08)	2.66 (2.26-3.14)	2.98 (2.53-3.52)
[45,50)	4.26 (3.66-4.95)	4.78 (4.11-5.57)	4.51 (3.87-5.25)	4.68 (4.03-5.44)	5.25 (4.51-6.11)
[50,55)	6.22 (5.38-7.19)	6.87 (5.93-7.95)	6.61 (5.71-7.65)	7.01 (6.07-8.10)	7.71 (6.66-8.92)
[55,60)	8.55 (7.42-9.84)	9.20 (7.98-10.61)	9.03 (7.83-10.42)	9.83 (8.53-11.32)	10.41 (9.02-12.01)
[60,65)	12.03 (10.46-13.82)	12.58 (10.93-14.47)	12.66 (10.99-14.58)	14.23 (12.38-16.36)	14.56 (12.64-16.77)
[65,70)	14.09 (12.25-16.19)	13.97 (12.13-16.09)	14.81 (12.86-17.07)	16.91 (14.71-19.44)	16.50 (14.31-19.03)
[70,75)	15.10 (13.13-17.36)	13.25 (11.45-15.32)	15.80 (13.70-18.22)	18.46 (16.05-21.24)	16.19 (13.97-18.76)
[75,80)	14.51 (12.57-16.75)	11.87 (10.21-13.81)	14.98 (12.94-17.34)	18.12 (15.69-20.92)	14.80 (12.69-17.25)

[80,85)	10.38 (8.83-12.19)	8.12 (6.86-9.61)	10.45 (8.87-12.31)	13.21 (11.24-15.52)	10.26 (8.65-12.17)
[85,90)	4.55 (3.59-5.77)	3.41 (2.67-4.34)	4.43 (3.49-5.63)	5.78 (4.56-7.32)	4.26 (3.34-5.44)
[90,100)	1.24 (0.72-2.13)	0.88 (0.51-1.52)	1.15 (0.67-1.98)	1.53 (0.89-2.62)	1.06 (0.61-1.83)
[100,115]	0.00 (0.00-0.00)	0.00 (0.00-0.00)	0.00 (0.00-0.00)	0.00 (0.00-0.00)	0.00 (0.00-0.00)
Vaccinated				RR (LCL-UCL)	RR (LCL-UCL)
No				1 (ref)	1 (ref)
Yes				0.17 (0.16-0.18)	0.17 (0.16-0.19)
Education		RR (LCL-UCL)			RR (LCL-UCL)
Primary		1 (ref)			1 (ref)
Secondary		0.80 (0.76-0.84)			0.79 (0.75-0.84)
Post-secondary		0.62 (0.58-0.66)			0.60 (0.56-0.64)
Unknown		0.98 (0.86-1.12)			0.94 (0.82-1.08)
Disposable Income Quartiles		RR (LCL-UCL)			RR (LCL-UCL)
1-24		1 (ref)			1 (ref)
25-49		0.95 (0.89-1.01)			0.98 (0.92-1.04)
50-74		0.97 (0.91-1.04)			1.03 (0.96-1.10)
75-100		0.93 (0.86-1.01)			0.98 (0.91-1.07)
Broad skill level		RR (LCL-UCL)			RR (LCL-UCL)
3,4		1 (ref)			1 (ref)
2		1.11 (1.03-1.20)			1.14 (1.06-1.23)
1		1.25 (1.11-1.41)			1.22 (1.08-1.37)
AF		0.45 (0.11-1.79)			0.45 (0.11-1.82)
X		1.40 (1.29-1.51)			1.36 (1.26-1.47)
Household type		RR (LCL-UCL)			RR (LCL-UCL)
Cohabitating		1 (ref)			1 (ref)
Single		1.12 (1.06-1.19)			0.96 (0.90-1.02)
Other		1.17 (1.09-1.25)			1.09 (1.01-1.17)
Accomodation type			RR (LCL-UCL)		RR (LCL-UCL)
House			1 (ref)		1 (ref)
Apartment			1.45 (1.37-1.54)		1.33 (1.25-1.42)
Special/student accomodation			0.80 (0.54-1.18)		0.76 (0.51-1.12)
Elderly care			1.70 (1.39-2.07)		1.55 (1.26-1.89)
Other accomodation			1.56 (1.32-1.84)		1.44 (1.22-1.71)
Living area density quartiles(person/sqm)			RR (LCL-UCL)		RR (LCL-UCL)
Q1			1 (ref)		1 (ref)
Q2			0.99 (0.92-1.07)		1.10 (1.02-1.19)
Q3			0.96 (0.89-1.03)		1.10 (1.01-1.19)
Q4			1.00 (0.93-1.08)		1.17 (1.06-1.28)

DeSO population density (person/sqkm)			RR (LCL-UCL)		RR (LCL-UCL)
(1,95.4]			1 (ref)		1 (ref)
(95.4,386]			1.09 (1.01-1.18)		1.15 (1.06-1.24)
(386,1.47e+03]			1.12 (1.04-1.21)		1.23 (1.14-1.33)
(1.47e+03,4.82e+03]			1.13 (1.04-1.22)		1.27 (1.16-1.38)
(4.82e+03,5.56e+04]			1.10 (1.01-1.21)		1.25 (1.14-1.38)
Region			RR (LCL-UCL)		RR (LCL-UCL)
East			1 (ref)		1 (ref)
Mid-west			0.84 (0.78-0.91)		0.81 (0.75-0.87)
North			0.90 (0.84-0.97)		0.87 (0.81-0.94)
South			0.52 (0.48-0.56)		0.49 (0.45-0.53)
South-east			0.71 (0.65-0.77)		0.68 (0.63-0.74)
West			0.85 (0.80-0.90)		0.81 (0.76-0.86)

* 3,4: Managers, professionals, technicians and associate professionals
2: Clerical, service, and sales workers, skilled agricultural, forestry and fishery worker, craft and related trade workers, and plant and machine operators, and assemblers
1: Elementary occupations
AF: Armed Forces
X: Not elsewhere classified

Supplementary Table 2: Association between country/region of origin and Covid-19 related mortality: RRs and 95% Cis by different model specification.

	Model 1: Sex, age, and region of origin	Model 2: Model 1 + SES	Model 3: Model 1 + living conditions	Model 4: Model 1 + vaccines	Model 5: Model 1 + SES + living conditions + vaccines
AIC:	247185	245073	239915	255128	245448
Residuals					
	RR (LCL-UCL)	RR (LCL-UCL)	RR (LCL-UCL)	RR (LCL-UCL)	RR (LCL-UCL)
Region of Origin					
Sweden	1 (ref)	1 (ref)	1 (ref)	1 (ref)	1 (ref)
Nordics wo Sweden	1.47 (1.39-1.56)	1.36 (1.28-1.44)	1.38 (1.30-1.46)	1.44 (1.36-1.53)	1.27 (1.19-1.34)
EU28 wo Nordics	1.22 (1.14-1.32)	1.16 (1.07-1.25)	1.15 (1.06-1.23)	1.15 (1.07-1.24)	1.03 (0.95-1.11)
Europe wo EU28	2.27 (2.09-2.46)	1.89 (1.73-2.06)	2.12 (1.95-2.30)	2.07 (1.90-2.24)	1.65 (1.51-1.80)
Middle East	2.68 (2.46-2.91)	2.04 (1.86-2.23)	2.27 (2.08-2.48)	2.47 (2.27-2.69)	1.67 (1.52-1.82)
Africa	3.07 (2.66-3.55)	2.26 (1.95-2.63)	2.51 (2.17-2.91)	2.75 (2.38-3.17)	1.80 (1.55-2.09)
Asia	2.26 (1.95-2.62)	1.83 (1.57-2.13)	2.06 (1.78-2.39)	2.10 (1.82-2.44)	1.57 (1.35-1.83)
North America	0.74 (0.51-1.07)	0.76 (0.53-1.10)	0.71 (0.49-1.02)	0.72 (0.50-1.04)	0.72 (0.50-1.03)
South America	1.79 (1.49-2.15)	1.52 (1.26-1.83)	1.47 (1.22-1.77)	1.63 (1.36-1.96)	1.19 (0.98-1.43)
Age					
[20,30)	1 (ref)	1 (ref)	1 (ref)	1 (ref)	1 (ref)
[30,35)	1.49 (0.90-2.47)	1.82 (1.10-3.01)	1.57 (0.95-2.60)	1.81 (1.09-2.99)	2.16 (0.91-5.15)
[35,40)	1.70 (1.04-2.79)	2.21 (1.35-3.62)	1.89 (1.15-3.11)	2.27 (1.39-3.73)	3.80 (1.58-9.16)
[40,45)	3.14 (2.02-4.88)	4.16 (2.68-6.47)	3.68 (2.37-5.74)	4.45 (2.86-6.93)	4.88 (2.04-11.68)
[45,50)	5.27 (3.51-7.92)	7.04 (4.68-10.59)	6.35 (4.21-9.56)	7.65 (5.08-11.52)	9.75 (4.19-22.68)
[50,55)	10.73 (7.33-15.70)	13.93 (9.51-20.41)	12.73 (8.66-18.70)	14.78 (10.07-21.70)	17.08 (7.46-39.08)
[55,60)	17.53 (12.09-25.42)	21.72 (14.98-31.50)	19.89 (13.67-28.94)	21.87 (15.05-31.78)	33.61 (14.88-75.91)
[60,65)	34.08 (23.68-49.04)	40.27 (27.97-57.98)	37.05 (25.65-53.53)	38.28 (26.60-55.08)	42.16 (29.81-59.65)
[65,70)	64.36 (44.92-92.22)	67.41 (47.00-96.68)	68.62 (47.70-98.71)	72.92 (50.89-104.48)	70.15 (49.78-98.85)
[70,75)	130.42 (91.31-186.27)	100.98 (70.45-144.74)	136.56 (95.23-195.82)	149.49 (104.66-213.51)	107.75 (76.52-151.74)
[75,80)	268.44 (188.16-382.96)	180.39 (125.87-258.53)	269.40 (188.07-385.90)	311.80 (218.56-444.82)	190.83 (135.51-268.72)
[80,85)	581.22 (407.60-828.80)	362.29 (252.85-519.10)	540.71 (377.64-774.19)	683.95 (479.64-975.29)	370.85 (263.44-522.05)
[85,90)	1236.44 (867.25-1762.78)	710.75 (496.05-1018.37)	992.58 (693.26-1421.14)	1452.55 (1018.85-2070.88)	664.20 (471.84-934.97)

[90,100)	2643.34 (1854.31-3768.13)	1389.59 (969.70-1991.28)	1650.11 (1152.25-2363.08)	3039.95 (2132.59-4333.36)	1064.10 (755.73-1498.28)
[100,115]	5559.48 (3811.66-8108.75)	2718.74 (1853.71-3987.44)	2563.01 (1747.69-3758.68)	5954.65 (4084.12-8681.89)	1506.16 (1043.81-2173.30)
Vaccinated				RR (LCL-UCL)	RR (LCL-UCL)
No				1 (ref)	1 (ref)
Yes				0.35 (0.34-0.36)	0.36 (0.35-0.37)
Education		RR (LCL-UCL)			RR (LCL-UCL)
Primary		1 (ref)			1 (ref)
Secondary		0.90 (0.87-0.93)			0.87 (0.84-0.90)
Post-secondary		0.72 (0.69-0.76)			0.66 (0.63-0.69)
Unknown		1.05 (0.96-1.15)			0.99 (0.91-1.08)
Disposable Income Quartiles		RR (LCL-UCL)			RR (LCL-UCL)
1-24		1 (ref)			1 (ref)
25-49		0.87 (0.83-0.90)			0.92 (0.88-0.96)
50-74		0.74 (0.70-0.79)			0.73 (0.69-0.78)
75-100		0.83 (0.77-0.90)			0.72 (0.67-0.78)
Broad skill level*		RR (LCL-UCL)			RR (LCL-UCL)
3,4		1 (ref)			1 (ref)
2		1.24 (1.10-1.41)			1.33 (1.18-1.51)
1		1.67 (1.36-2.05)			1.67 (1.36-2.06)
AF		0.00 (0.00-0.00)			0.00 (0.00-0.00)
X		2.56 (2.28-2.88)			2.36 (2.10-2.65)
Household type		RR (LCL-UCL)			RR (LCL-UCL)
Cohabitating		1 (ref)			1 (ref)
Single		1.46 (1.40-1.52)			0.82 (0.78-0.86)
Other		1.65 (1.56-1.75)			1.73 (1.61-1.85)
Accomodation type			RR (LCL-UCL)		RR (LCL-UCL)
House			1 (ref)		1 (ref)
Apartment			1.59 (1.52-1.66)		1.56 (1.49-1.63)
Special/student accomodation			3.49 (2.58-4.74)		3.45 (2.54-4.68)
Elderly care			6.89 (6.50-7.29)		7.27 (6.82-7.74)
Other accomodation			2.08 (1.89-2.29)		2.08 (1.89-2.29)
Living area density quartiles(person/sqm)			RR (LCL-UCL)		RR (LCL-UCL)
1-24			1 (ref)		1 (ref)
25-49			1.02 (0.95-1.09)		1.44 (1.33-1.57)
50-74			0.94 (0.87-1.01)		1.40 (1.29-1.53)

75-100			1.63 (1.52-1.75)		2.65 (2.42-2.91)
<u>DeSO population density (person/spkm)</u>			<u>RR (LCL-UCL)</u>		<u>RR (LCL-UCL)</u>
(1,95.4]			1 (ref)		1 (ref)
(95.4,386]			1.07 (1.01-1.14)		1.11 (1.05-1.18)
(386,1.47e +03]			1.12 (1.06-1.19)		1.21 (1.14-1.28)
(1.47e+03,4.82e+03]			1.19 (1.13-1.26)		1.37 (1.29-1.45)
(4.82e+03,5.56e+04]			1.24 (1.16-1.32)		1.46 (1.37-1.55)
<u>Region</u>			<u>RR (LCL-UCL)</u>		<u>RR (LCL-UCL)</u>
East			1 (ref)		1 (ref)
Mid-west			0.70 (0.66-0.73)		0.66 (0.63-0.69)
North			0.78 (0.74-0.82)		0.74 (0.70-0.78)
South			0.71 (0.67-0.74)		0.67 (0.64-0.70)
South-east			0.62 (0.58-0.65)		0.59 (0.55-0.62)
West			0.76 (0.73-0.79)		0.73 (0.70-0.76)

* 3,4: Managers, professionals, technicians and associate professionals
2: Clerical, service, and sales workers, skilled agricultural, forestry and fishery worker, craft and related trade workers, and plant and machine operators, and assemblers
1: Elementary occupations
AF: Armed Forces
X: Not elsewhere classified

Supplementary Table 3: Percentage change of excess mortality for the subsequent models.

**ICU**	Δ(M2,M1)	Δ(M3,M1)	Δ(M4,M1)	Δ(M5,M1)
	23,2	18,7	10,3	51,5
	19,0	14,3	33,6	58,4
	19,7	15,1	16,3	42,7
	21,4	22,1	11,9	43,7
	26,7	31,2	14,3	54,8
	20,5	18,7	8,1	36,6
	15,0	-23,7	-19,6	-80,2
	11,8	26,3	11,1	40,3
**Mortality**	Δ(M2,M1)	Δ(M3,M1)	Δ(M4,M1)	Δ(M5,M1)
	23,7	23,0	6,5	46,7
	30,5	46,8	30,8	99,9
	30,2	14,4	16,1	51,9
	38,5	26,8	12,4	63,9
	39,4	29,6	15,8	64,6
	34,1	18,6	12,4	58,8
	8,3	-16,3	-7,7	-14,6
	34,9	45,6	20,1	83,0

Supplementary Table 4: Association between country/region of origin and Covid-19 related ICU admission for the seven time periods (the four waves of elevated Covid-19 infections and three phases of low Covid-19 infections): RRs and 95% Cis for M1, M4, and M5 model specifications*.

	M1	M4	M5
AIC	4866.9	5818.5	106916
Region of origin : period			
Sweden:Wave 1	1.00 (ref)	1.00 (ref)	1.00 (ref)
Nordics w/o Sweden:Wave 1	1.45 (1.18-1.78)	1.42 (1.12-1.79)	1.27 (1.02-1.58)
EU28/EEA w/o Nordics:Wave 1	1.41 (1.09-1.82)	1.43 (1.10-1.86)	1.31 (1.04-1.66)
Europe w/o EU28/EEA:Wave 1	4.10 (3.44-4.90)	4.22 (3.45-5.17)	3.37 (2.87-3.97)
Middle East:Wave 1	6.18 (5.15-7.41)	6.50 (5.39-7.85)	4.81 (4.24-5.47)
Africa:Wave 1	8.21 (6.65-10.15)	8.77 (7.08-10.87)	5.73 (4.83-6.80)
Asia:Wave 1	4.29 (3.29-5.58)	4.60 (3.50-6.04)	3.65 (2.98-4.47)
North America:Wave 1	1.24 (0.55-2.82)	1.28 (0.56-2.92)	1.33 (0.55-3.19)
South America:Wave 1	5.82 (4.63-7.31)	6.03 (4.71-7.70)	4.67 (3.70-5.89)
Sweden:phase1	1.00 (ref)	1.00 (ref)	1.00 (ref)
Nordics w/o Sweden:phase1	1.71 (1.03-2.83)	1.67 (0.99-2.82)	1.23 (0.57-2.63)
EU28/EEA w/o Nordics:phase1	1.22 (0.51-2.92)	1.24 (0.52-2.91)	1.02 (0.42-2.47)
Europe w/o EU28/EEA:phase1	3.92 (2.56-6.01)	4.03 (2.62-6.21)	3.37 (2.09-5.44)
Middle East:phase1	3.28 (2.00-5.39)	3.46 (2.14-5.58)	2.70 (1.80-4.05)
Africa:phase1	3.79 (1.79-8.02)	4.05 (1.95-8.37)	2.13 (0.95-4.75)
Asia:phase1	1.93 (0.73-5.13)	2.07 (0.79-5.42)	1.69 (0.68-4.22)
North America:phase1	0.00 (0.00-0.00)	0.00 (0.00-0.00)	0.00 (0.00-0.00)
South America:phase1	2.29 (0.92-5.72)	2.37 (0.95-5.94)	1.86 (0.56-6.20)
Sweden:Wave 2	1.00 (ref)	1.00 (ref)	1.00 (ref)
Nordics w/o Sweden:Wave 2	1.71 (1.22-2.41)	1.68 (1.16-2.44)	1.49 (0.96-2.30)
EU28/EEA w/o Nordics:Wave 2	1.82 (1.13-2.96)	1.85 (1.17-2.93)	1.69 (1.07-2.67)
Europe w/o EU28/EEA:Wave 2	3.64 (2.56-5.18)	3.74 (2.61-5.35)	3.01 (2.23-4.07)
Middle East:Wave 2	4.19 (3.08-5.70)	4.40 (3.31-5.84)	3.27 (2.64-4.07)
Africa:Wave 2	2.79 (1.86-4.17)	2.97 (2.03-4.36)	2.04 (1.37-3.02)
Asia:Wave 2	3.58 (2.23-5.75)	3.83 (2.42-6.08)	3.04 (2.03-4.55)
North America:Wave 2	0.00 (0.00-0.00)	0.00 (0.00-0.00)	0.00 (0.00-0.00)
South America:Wave 2	3.43 (2.20-5.34)	3.54 (2.28-5.51)	2.77 (1.64-4.67)
Sweden:Wave 3	1.00 (ref)	1.00 (ref)	1.00 (ref)
Nordics w/o Sweden:Wave 3	1.49 (1.04-2.13)	1.48 (1.01-2.17)	1.31 (0.85-2.02)
EU28/EEA w/o Nordics:Wave 3	1.20 (0.74-1.96)	1.09 (0.69-1.73)	1.01 (0.63-1.61)
Europe w/o EU28/EEA:Wave 3	2.47 (1.90-3.21)	2.17 (1.62-2.89)	1.76 (1.29-2.41)
Middle East:Wave 3	3.42 (2.59-4.52)	2.96 (2.28-3.84)	2.23 (1.79-2.76)
Africa:Wave 3	2.38 (1.56-3.63)	2.06 (1.38-3.05)	1.40 (0.95-2.05)
Asia:Wave 3	3.93 (2.49-6.22)	3.45 (2.21-5.36)	2.63 (1.79-3.87)
North America:Wave 3	0.94 (0.14-6.44)	0.85 (0.13-5.69)	0.86 (0.11-6.68)
South America:Wave 3	2.81 (1.85-4.26)	2.51 (1.62-3.87)	1.86 (1.10-3.14)

Sweden:phase2	1.00 (ref)	1.00 (ref)	1.00 (ref)
Nordics w/o Sweden:phase2	1.51 (0.98-2.33)	1.30 (0.85-1.99)	1.13 (0.61-2.09)
EU28/EEA w/o Nordics:phase2	2.76 (1.76-4.34)	1.48 (1.00-2.18)	1.41 (0.85-2.34)
Europe w/o EU28/EEA:phase2	7.46 (6.08-9.15)	4.05 (3.27-5.02)	3.44 (2.55-4.63)
Middle East:phase2	7.24 (5.61-9.35)	4.11 (3.38-5.00)	3.22 (2.59-4.01)
Africa:phase2	6.18 (4.40-8.68)	3.10 (2.21-4.36)	2.13 (1.36-3.33)
Asia:phase2	4.19 (1.91-9.21)	2.65 (1.26-5.56)	2.22 (1.32-3.73)
North America:phase2	0.00 (0.00-0.00)	0.00 (0.00-0.00)	0.00 (0.00-0.00)
South America:phase2	1.66 (0.69-4.02)	1.04 (0.42-2.54)	0.82 (0.28-2.41)
Sweden:Wave 4	1.00 (ref)	1.00 (ref)	1.00 (ref)
Nordics w/o Sweden:Wave 4	1.85 (1.31-2.60)	1.54 (1.06-2.23)	1.39 (0.88-2.18)
EU28/EEA w/o Nordics:Wave 4	2.06 (1.36-3.14)	1.08 (0.72-1.61)	1.02 (0.64-1.61)
Europe w/o EU28/EEA:Wave 4	2.78 (1.94-3.99)	1.57 (1.14-2.16)	1.34 (0.96-1.87)
Middle East:Wave 4	2.49 (1.76-3.51)	1.61 (1.23-2.12)	1.25 (0.96-1.63)
Africa:Wave 4	2.40 (1.36-4.22)	1.41 (0.90-2.22)	1.02 (0.64-1.63)
Asia:Wave 4	2.10 (1.16-3.81)	1.52 (0.90-2.57)	1.23 (0.74-2.05)
North America:Wave 4	1.02 (0.11-9.02)	0.67 (0.08-5.93)	0.69 (0.06-7.56)
South America:Wave 4	2.32 (1.34-4.03)	1.55 (0.87-2.76)	1.23 (0.63-2.38)
Sweden:phase3	1.00 (ref)	1.00 (ref)	1.00 (ref)
Nordics w/o Sweden:phase3	0.00 (0.00-0.00)	0.00 (0.00-0.00)	0.00 (0.00-0.00)
EU28/EEA w/o Nordics:phase3	0.00 (0.00-0.00)	0.00 (0.00-0.00)	0.00 (0.00-0.00)
Europe w/o EU28/EEA:phase3	1.57 (0.26-9.58)	0.89 (0.15-5.17)	0.74 (0.14-3.92)
Middle East:phase3	0.00 (0.00-0.00)	0.00 (0.00-0.00)	0.00 (0.00-0.00)
Africa:phase3	3.19 (0.54-18.90)	1.92 (0.33-11.11)	1.35 (0.25-7.21)
Asia:phase3	0.00 (0.00-0.00)	0.00 (0.00-0.00)	0.00 (0.00-0.00)
North America:phase3	0.00 (0.00-0.00)	0.00 (0.00-0.00)	0.00 (0.00-0.00)
South America:phase3	0.00 (0.00-0.00)	0.00 (0.00-0.00)	0.00 (0.00-0.00)
Sex			
Male	2.33 (2.20-2.48)	2.29 (2.15-2.44)	2.31 (2.20-2.42)
Age category			
[20,30)	1(ref)	1(ref)	1(ref)
[30,35)	1.33 (0.97-1.82)	1.35 (0.99-1.83)	1.40 (1.16-1.70)
[35,40)	1.64 (1.22-2.21)	1.71 (1.29-2.25)	1.94 (1.62-2.32)
[40,45)	2.57 (1.94-3.41)	2.72 (2.08-3.55)	3.08 (2.61-3.64)
[45,50)	4.40 (3.39-5.71)	4.76 (3.69-6.12)	5.34 (4.58-6.21)
[50,55)	6.43 (5.02-8.23)	7.11 (5.54-9.13)	7.90 (6.83-9.13)
[55,60)	8.83 (6.91-11.28)	10.00 (7.79-12.82)	10.64 (9.23-12.26)
[60,65)	12.41 (9.79-15.73)	14.64 (11.51-18.61)	15.13 (13.15-17.41)
[65,70)	14.52 (11.45-18.41)	17.56 (13.79-22.36)	17.21 (14.94-19.83)
[70,75)	15.53 (12.19-19.78)	19.43 (15.17-24.89)	17.13 (14.80-19.83)
[75,80)	14.88 (11.55-19.18)	19.47 (14.96-25.34)	15.81 (13.57-18.41)
[80,85)	10.58 (7.91-14.15)	14.62 (11.01-19.40)	11.22 (9.47-13.30)

[85,90)	4.59 (2.83-7.44)	6.57 (4.43-9.76)	4.96 (3.88-6.34)
[90,100)	1.23 (0.69-2.19)	1.79 (1.01-3.20)	1.30 (0.75-2.25)
[100,115]	0.00 (0.00-0.00)	0.00 (0.00-0.00)	0.00 (0.00-0.00)
Vaccinated			
No		1(ref)	1(ref)
Yes		0.15 (0.12-0.18)	0.16 (0.14-0.18)
Education			
Primary			1(ref)
Secondary			0.80 (0.76-0.84)
Post-secondary			0.61 (0.57-0.65)
Unknown			0.80 (0.69-0.92)
Disposable income			
0-24			1(ref)
25-49			0.99 (0.93-1.06)
50-74			1.03 (0.96-1.11)
75-100			0.98 (0.90-1.06)
Broad skill level			
3,4			1(ref)
2			1.15 (1.07-1.24)
1			1.25 (1.11-1.41)
AF			0.47 (0.12-1.86)
X			1.37 (1.27-1.48)
Household			
Partner			1(ref)
Single			0.96 (0.90-1.03)
Other			1.11 (1.02-1.19)
Building type			
House			1(ref)
Apartment			1.34 (1.26-1.43)
Special/student accommodation			0.83 (0.57-1.21)
Elderly care			1.65 (1.34-2.02)
Other accomodation			0.58 (0.39-0.86)
Living area per person quartiles			
0-24			1(ref)
25-49			1.09 (1.01-1.18)
50-74			1.08 (1.00-1.17)
75-100			1.14 (1.04-1.26)
DeSO population density			
[0,95.4]			1(ref)
(95.4,386]			1.15 (1.06-1.24)
(386,1.47e+03]			1.21 (1.12-1.31)
(1.47e+03,4.82e+03]			1.24 (1.14-1.35)
(4.82e+03,5.56e+04]			1.23 (1.12-1.36)
Region			
East			1(ref)

Mid-west			0.82 (0.76-0.88)
North			0.88 (0.81-0.95)
South			0.49 (0.45-0.53)
South-east			0.68 (0.63-0.74)
West			0.81 (0.77-0.87)

* M1: model adjusted for age and sex.
M4: model adjusted for age, sex and vaccination status.
M5: model adjusted for age, sex, socioeconomic and living conditions and vaccination status.

Supplementary Table 5: Association between country/region of origin and Covid-19 related mortality for the seven time periods (the four waves of elevated Covid-19 infections and three phases of low Covid-19 infections): RRs and 95% Cis for M1, M4, and M5 model specifications*.

	M1	M4	M5
AIC	164441	163269	140916
Sweden:Wave 1	1.00 (ref)	1.00 (ref)	1.00 (ref)
Nordics w/o Sweden:Wave 1	1.59 (1.40-1.81)	1.59 (1.39-1.81)	1.36 (1.21-1.54)
EU28/EEA w/o Nordics:Wave 1	1.27 (1.08-1.48)	1.27 (1.09-1.47)	1.18 (1.02-1.38)
Europe w/o EU28/EEA:Wave 1	2.34 (2.01-2.72)	2.36 (2.03-2.75)	1.77 (1.48-2.12)
Middle East:Wave 1	4.19 (3.53-4.97)	4.28 (3.60-5.09)	2.46 (2.07-2.93)
Africa:Wave 1	5.83 (4.51-7.54)	6.02 (4.61-7.85)	3.40 (2.64-4.39)
Asia:Wave 1	2.60 (1.76-3.84)	2.68 (1.81-3.96)	2.12 (1.58-2.84)
North America:Wave 1	1.08 (0.68-1.71)	1.08 (0.68-1.71)	1.26 (0.71-2.22)
South America:Wave 1	2.40 (1.93-2.99)	2.44 (1.95-3.06)	1.82 (1.31-2.52)
Sweden:phase1	1.00 (ref)	1.00 (ref)	1.00 (ref)
Nordics w/o Sweden:phase1	1.09 (0.76-1.55)	1.09 (0.75-1.57)	0.95 (0.57-1.55)
EU28/EEA w/o Nordics:phase1	0.97 (0.55-1.74)	0.97 (0.54-1.74)	1.00 (0.54-1.84)
Europe w/o EU28/EEA:phase1	2.24 (1.28-3.92)	2.27 (1.29-4.00)	2.00 (1.03-3.88)
Middle East:phase1	1.61 (0.70-3.70)	1.65 (0.71-3.83)	1.06 (0.41-2.70)
Africa:phase1	1.63 (0.37-7.28)	1.68 (0.37-7.70)	0.70 (0.09-5.74)
Asia:phase1	1.89 (0.47-7.51)	1.94 (0.48-7.88)	1.85 (0.49-7.04)
North America:phase1	0.00 (0.00-0.00)	0.00 (0.00-0.00)	0.00 (0.00-0.00)
South America:phase1	0.79 (0.10-6.13)	0.80 (0.10-6.36)	0.68 (0.08-6.10)
Sweden:Wave 2	1.00 (ref)	1.00 (ref)	1.00 (ref)
Nordics w/o Sweden:Wave 2	1.50 (1.24-1.82)	1.50 (1.22-1.84)	1.30 (1.04-1.63)
EU28/EEA w/o Nordics:Wave 2	1.10 (0.84-1.44)	1.09 (0.84-1.42)	1.00 (0.74-1.35)
Europe w/o EU28/EEA:Wave 2	2.03 (1.59-2.59)	2.03 (1.56-2.64)	1.58 (1.10-2.27)
Middle East:Wave 2	1.57 (1.09-2.24)	1.58 (1.08-2.30)	0.91 (0.61-1.36)
Africa:Wave 2	1.45 (0.79-2.65)	1.47 (0.78-2.75)	1.19 (0.63-2.25)
Asia:Wave 2	1.37 (0.60-3.13)	1.39 (0.60-3.25)	1.19 (0.60-2.36)
North America:Wave 2	0.74 (0.28-2.01)	0.74 (0.27-2.04)	0.59 (0.14-2.51)
South America:Wave 2	1.09 (0.65-1.83)	1.09 (0.64-1.88)	0.84 (0.37-1.88)
Sweden:Wave 3	1.00 (ref)	1.00 (ref)	1.00 (ref)
Nordics w/o Sweden:Wave 3	1.37 (1.05-1.78)	1.31 (1.01-1.72)	1.17 (0.90-1.53)
EU28/EEA w/o Nordics:Wave 3	1.62 (1.26-2.08)	1.45 (1.05-1.99)	1.37 (1.00-1.89)
Europe w/o EU28/EEA:Wave 3	2.67 (2.01-3.54)	2.15 (1.53-3.03)	1.77 (1.19-2.62)
Middle East:Wave 3	3.49 (2.68-4.54)	2.67 (1.88-3.80)	2.02 (1.38-2.94)
Africa:Wave 3	3.59 (2.05-6.27)	2.57 (1.37-4.82)	1.90 (0.99-3.61)
Asia:Wave 3	5.61 (2.75-11.42)	4.32 (2.00-9.31)	3.40 (1.81-6.38)
North America:Wave 3	0.68 (0.16-2.80)	0.65 (0.14-2.93)	0.69 (0.12-4.04)
South America:Wave 3	3.26 (1.91-5.55)	2.54 (1.38-4.67)	1.94 (0.89-4.23)
Sweden:phase2	1.00 (ref)	1.00 (ref)	1.00 (ref)

Nordics w/o Sweden:phase2	1.34 (1.09-1.64)	1.25 (1.02-1.53)	1.14 (0.78-1.66)
EU28/EEA w/o Nordics:phase2	1.28 (0.80-2.04)	1.05 (0.70-1.56)	1.04 (0.66-1.66)
Europe w/o EU28/EEA:phase2	4.10 (2.93-5.73)	3.03 (2.22-4.15)	2.72 (1.75-4.26)
Middle East:phase2	6.68 (4.79-9.31)	5.04 (3.64-6.99)	4.14 (2.78-6.14)
Africa:phase2	6.31 (3.19-12.44)	4.25 (2.20-8.22)	3.32 (1.57-7.03)
Asia:phase2	3.72 (1.44-9.63)	2.87 (1.19-6.93)	2.55 (1.05-6.21)
North America:phase2	0.89 (0.08-10.15)	0.79 (0.08-8.17)	0.89 (0.07-10.75)
South America:phase2	1.57 (0.49-4.97)	1.14 (0.36-3.58)	0.92 (0.23-3.66)
Sweden:Wave 4	1.00 (ref)	1.00 (ref)	1.00 (ref)
Nordics w/o Sweden:Wave 4	1.34 (1.03-1.73)	1.26 (1.04-1.53)	1.13 (0.91-1.41)
EU28/EEA w/o Nordics:Wave 4	1.17 (0.86-1.60)	0.97 (0.81-1.16)	0.93 (0.70-1.24)
Europe w/o EU28/EEA:Wave 4	2.13 (1.62-2.80)	1.63 (1.34-1.99)	1.39 (0.98-1.97)
Middle East:Wave 4	1.73 (1.15-2.61)	1.40 (1.06-1.84)	1.14 (0.79-1.64)
Africa:Wave 4	1.78 (0.91-3.49)	1.31 (0.75-2.27)	1.00 (0.53-1.89)
Asia:Wave 4	1.35 (0.55-3.33)	1.10 (0.52-2.33)	0.95 (0.47-1.94)
North America:Wave 4	0.39 (0.07-2.08)	0.35 (0.07-1.69)	0.38 (0.07-2.21)
South America:Wave 4	1.56 (0.80-3.03)	1.19 (0.70-2.01)	0.88 (0.40-1.94)
Sweden:phase3	1.00 (ref)	1.00 (ref)	1.00 (ref)
Nordics w/o Sweden:phase3	1.82 (1.16-2.87)	1.72 (1.18-2.53)	1.45 (0.88-2.39)
EU28/EEA w/o Nordics:phase3	1.20 (0.68-2.14)	1.00 (0.61-1.62)	0.92 (0.45-1.90)
Europe w/o EU28/EEA:phase3	0.79 (0.22-2.80)	0.61 (0.17-2.09)	0.51 (0.13-2.03)
Middle East:phase3	0.45 (0.07-3.13)	0.37 (0.06-2.46)	0.29 (0.04-2.04)
Africa:phase3	0.00 (0.00-0.00)	0.00 (0.00-0.00)	0.00 (0.00-0.00)
Asia:phase3	1.24 (0.14-10.84)	1.02 (0.12-8.73)	0.00 (0.00-0.00)
North America:phase3	0.00 (0.00-0.00)	0.00 (0.00-0.00)	0.00 (0.00-0.00)
South America:phase3	0.00 (0.00-0.00)	0.00 (0.00-0.00)	0.00 (0.00-0.00)
Sex			
Female	1(ref)	1(ref)	1(ref)
Male	1.76 (1.65-1.88)	2.29 (2.15-2.44)	2.23 (2.16-2.31)
Age category			
[20,30)	1(ref)	1(ref)	1(ref)
[30,35)	1.45 (0.84-2.53)	1.47 (0.90-2.40)	2.07 (1.21-3.52)
[35,40)	1.66 (1.00-2.76)	1.70 (1.09-2.65)	2.78 (1.65-4.68)
[40,45)	3.06 (1.93-4.85)	3.17 (2.11-4.77)	5.04 (3.13-8.09)
[45,50)	5.12 (3.37-7.79)	5.39 (3.77-7.69)	9.18 (5.94-14.19)
[50,55)	10.43 (6.56-16.58)	11.10 (7.51-16.39)	18.10 (12.03-27.23)
[55,60)	17.04 (11.40-25.49)	18.33 (13.14-25.57)	26.65 (17.89-39.70)
[60,65)	33.10 (21.81-50.25)	36.25 (25.64-51.24)	48.68 (32.91-72.00)
[65,70)	62.45 (42.27-92.25)	69.01 (49.79-95.64)	79.95 (54.24-117.83)
[70,75)	126.32 (87.26-182.87)	141.40 (103.96-192.33)	123.18 (83.64-181.40)

[75,80)	259.37 (180.36-372.98)	295.06 (217.35-400.55)	216.59 (147.07-318.97)
[80,85)	558.87 (390.07-800.72)	648.65 (479.05-878.29)	422.00 (286.62-621.34)
[85,90)	1177.08 (818.42-1692.90)	1385.17 (1022.74-1876.03)	766.18 (520.35-1128.14)
[90,100)	2465.55 (1707.08-3561.00)	2935.95 (2166.13-3979.35)	1266.71 (859.86-1866.06)
[100,115]	4950.78 (3364.58-7284.77)	5879.60 (4177.22-8275.75)	1863.84 (1232.90-2817.66)
Vaccinated			
No		1(ref)	1(ref)
Yes		0.33 (0.28-0.40)	0.31 (0.29-0.33)
Education			
Primary			1(ref)
Secondary			0.88 (0.85-0.91)
Post-secondary			0.67 (0.64-0.70)
Unknown			0.47 (0.42-0.54)
Disposable income			
0-24			1(ref)
25-49			0.91 (0.87-0.95)
50-74			0.73 (0.68-0.78)
75-100			0.71 (0.66-0.77)
Broad skill level			
3,4			1(ref)
2			1.31 (1.15-1.48)
1			1.68 (1.36-2.06)
AF			0.00 (0.00-0.00)
X			2.28 (2.02-2.56)
Household			
Partner			1(ref)
Single			0.78 (0.74-0.83)
Other			1.71 (1.59-1.83)
Building type			
House			1(ref)
Apartment			1.59 (1.51-1.67)
Special/student accommodation			3.29 (2.41-4.48)
Elderly care			7.76 (7.24-8.31)
Other accomodation			1.87 (1.66-2.12)
Living area per person quartiles			
0-24			1(ref)
25-49			1.44 (1.32-1.57)
50-74			1.38 (1.26-1.51)
75-100			2.62 (2.38-2.89)
DeSO population density			

[0,95.4]			1(ref)
(95.4,386]			1.08 (1.02-1.15)
(386,1.47e+03]			1.15 (1.08-1.22)
(1.47e+03,4.82e+03]			1.29 (1.21-1.37)
(4.82e+03,5.56e+04]			1.37 (1.29-1.47)
Region			
East			1(ref)
Mid-west			0.66 (0.63-0.70)
North			0.75 (0.71-0.79)
South			0.68 (0.64-0.71)
South-east			0.59 (0.55-0.62)
West			0.74 (0.70-0.77)

* M1: model adjusted for age and sex.
M4: model adjusted for age, sex and vaccination status.
M5: model adjusted for age, sex, socioeconomic and living conditions and vaccination status.
