# Supplementary material for: Inequalities in COVID-19 severe morbidity and mortality by country of birth in Sweden
Source: Nat Commun. 2023 Aug 15;14:4919. doi: 10.1038/s41467-023-40568-4 (PMC10427621; doi:10.1038/s41467-023-40568-4)
Supplement: Supplementary file 3 — Reporting Summary [file 41467_2023_40568_MOESM3_ESM.pdf]

nature portfolio

Corresponding author(s): Mikael Rostila

Last updated by author(s): Jul 7, 2023

Reporting Summary

Nature Portfolio wishes to improve the reproducibility of the work that we publish. This form provides structure for consistency and transparency in reporting. For further information on Nature Portfolio policies, see our Editorial Policies and the Editorial Policy Checklist.

Statistics

For all statistical analyses, confirm that the following items are present in the figure legend, table legend, main text, or Methods section.

n/a	Confirmed	
☐	☒	The exact sample size (*n*) for each experimental group/condition, given as a discrete number and unit of measurement
☐	☒	A statement on whether measurements were taken from distinct samples or whether the same sample was measured repeatedly
☐	☒	The statistical test(s) used AND whether they are one- or two-sided<br>*Only common tests should be described solely by name; describe more complex techniques in the Methods section.*
☐	☒	A description of all covariates tested
☒	☐	A description of any assumptions or corrections, such as tests of normality and adjustment for multiple comparisons
☐	☒	A full description of the statistical parameters including central tendency (e.g. means) or other basic estimates (e.g. regression coefficient) AND variation (e.g. standard deviation) or associated estimates of uncertainty (e.g. confidence intervals)
☒	☐	For null hypothesis testing, the test statistic (e.g. *F*, *t*, *r*) with confidence intervals, effect sizes, degrees of freedom and *P* value noted<br>*Give P values as exact values whenever suitable.*
☒	☐	For Bayesian analysis, information on the choice of priors and Markov chain Monte Carlo settings
☒	☐	For hierarchical and complex designs, identification of the appropriate level for tests and full reporting of outcomes
☒	☐	Estimates of effect sizes (e.g. Cohen's *d*, Pearson's *r*), indicating how they were calculated

Our web collection on statistics for biologists contains articles on many of the points above.

Software and code

Policy information about availability of computer code

Data collection	No software was used in data collection
Data analysis	The software used for data analyses is R version 4.2.2. The custom R scripts are available in Zenodo under Creative Commons license, doi:10.5281/zenodo.8119347

For manuscripts utilizing custom algorithms or software that are central to the research but not yet described in published literature, software must be made available to editors and reviewers. We strongly encourage code deposition in a community repository (e.g. GitHub). See the Nature Portfolio guidelines for submitting code & software for further information.

Data

Policy information about availability of data

All manuscripts must include a data availability statement. This statement should provide the following information, where applicable:

- Accession codes, unique identifiers, or web links for publicly available datasets
- A description of any restrictions on data availability
- For clinical datasets or third party data, please ensure that the statement adheres to our policy

The data that support the findings of this study are available from The Swedish Public Health Agency but restrictions apply due to the sensitive nature of individual health data. The research question needs ethical approval from the ethical review agency (Etikprövningsmyndigheten) and then a data request can be sent to Registerhantering@folkhalsomyndigheten.se. The timeframe for data access is up to six months, and data must be securely stored and handled in Sweden. The data

is linked from the Total Population Register, the Longitudinal Integrated Database for Health Insurance and Labor Market Studies (LISA), the Dwelling Units Register, the national notifiable disease registry (SmiNet), the Swedish Intensive Care Register, the Cause of Death Register, and the National Vaccination Register. The registers are linked via pseudonymized personal identification number. Data are however also available from the authors upon reasonable request and with permission of The Swedish Public Health Agency.

Human research participants

Policy information about studies involving human research participants and Sex and Gender in Research.

Reporting on sex and gender	Sex was used as a covariate.
Population characteristics	The population was characterized by information available in administrative registers. The main exposure was country of birth grouped into country/regions. Additional information on age, sex, income, education, occupation, household composition, housing characteristics such as type of dwelling and living area per person, as well as neighborhood population density and region of residence was used for additional adjustments.
Recruitment	N/A
Ethics oversight	The Swedish ethical review board approved this study (Dnr. 2021-05754-02)

Note that full information on the approval of the study protocol must also be provided in the manuscript.

Field-specific reporting

Please select the one below that is the best fit for your research. If you are not sure, read the appropriate sections before making your selection.

☐ Life sciences ☒ Behavioural & social sciences ☐ Ecological, evolutionary & environmental sciences

For a reference copy of the document with all sections, see nature.com/documents/nr-reporting-summary-flat.pdf

Behavioural & social sciences study design

All studies must disclose on these points even when the disclosure is negative.

Study description	The study is a quantitative observational study. Linked total population registers are used in time-to-event analyses estimating the relative risks between covid-19 mortality and ICU events and country/region of origin in Sweden.
Research sample	The total adult (20+ years) population residing in Sweden at the end of 2019. The sample is representative since it encompasses all individuals considered in the study. Since the total population is available in the registers we used the total population to avoid any sampling errors.
Sampling strategy	The study population is the total adult population in Sweden, therefore no sampling strategy is used. The whole sample of the total population is used in the study.
Data collection	The data was obtained from Statistics Sweden, the National Board of Health and Welfare, and the Public Health Agency, and was linked with the use of a pseudonymized personal identification number. The key is held at Statistics Sweden. After an approval from the Swedish ethical review board a data request was sent to the Public Health Agency where their data request process (including a lawyer judgement) is undergone. After that the data is transferred by a secure protocol. Since these are administrative registers the researchers do not themselves collect the data, rather it is transferred to the researcher after it is collected. The study participanas are completely unaware of the study hypothesis tested.
Timing	The data ranges from 1st of March 2020 to 1 of June 2022.
Data exclusions	1131 individuals with unknown country of birth, 13202 with missing DeSO neighborhood, and 83 with missing disposable income were excluded from the data set.
Non-participation	No participants declined to be a part of the study since these are pseudonymized administrative registers.
Randomization	This is an observational study where covariates are controlled for by estimating multivariate regression models (Poisson).

Reporting for specific materials, systems and methods

We require information from authors about some types of materials, experimental systems and methods used in many studies. Here, indicate whether each material, system or method listed is relevant to your study. If you are not sure if a list item applies to your research, read the appropriate section before selecting a response.

Materials & experimental systems

n/a	Involved in the study
☒	☐ Antibodies
☒	☐ Eukaryotic cell lines
☒	☐ Palaeontology and archaeology
☒	☐ Animals and other organisms
☒	☐ Clinical data
☒	☐ Dual use research of concern

Methods

n/a	Involved in the study
☒	☐ ChIP-seq
☒	☐ Flow cytometry
☒	☐ MRI-based neuroimaging
